# Modifiable Factors Associated With Chronic Pain 1 Year After Operative Management of Distal Radius Fractures: A Secondary Analysis of a Randomized Clinical Trial

**DOI:** 10.1001/jamanetworkopen.2020.28929

**Published:** 2020-12-18

**Authors:** Alfred P. Yoon, Chang Wang, Kelly A. Speth, Lu Wang, Kevin C. Chung, for The WRIST Group

**Affiliations:** 1 Section of Plastic Surgery, Department of Surgery, University of Michigan Medical School, Ann Arbor; 2 Department of Biostatistics, School of Public Health, University of Michigan, Ann Arbor

## Abstract

**Importance:**

Despite appropriate treatment, many patients who sustain distal radius fractures (DRFs) report persistent wrist pain. Chronic musculoskeletal pain is among the leading health problems in the elderly population associated with significant personal and societal burden.

**Objective:**

To identify modifiable preoperative factors that are significantly associated with developing chronic pain.

**Design, Setting, and Participants:**

This is a secondary analysis of the Wrist and Radius Injury Surgical Trial (WRIST), a randomized multicenter clinical trial of 24 study sites in the United States, Canada, and Singapore that enrolled patients from April 10, 2012, to December 31, 2016. Adults older than 60 years who sustained closed extra-articular DRFs, were treated operatively, and completed 12-month Michigan Hand Outcomes Questionnaires (MHQs) were included in this study. Analysis was conducted from September to December 2019.

**Interventions:**

Volar locking plate internal fixation, external fixation, or percutaneous pinning.

**Main Outcomes and Measures:**

12-month MHQ pain domain score. Inverse probability weighted logistic regression was used to identify factors associated with of chronic pain.

**Results:**

A total of 146 patients with DRF who were treated operatively and had 12-month MHQ scores met inclusion criteria. The mean (SD) patient age was 68.9 (7.2) years, 128 (87.6%) were women, and 93 (63.7%) were retired. Chronic pain was present in 87 patients (59.6%) and absent in 59 patients (40.4%) at 1-year follow-up. A 1-week delay in surgery was associated with more than triple the odds of developing chronic pain (odds ratio [OR], 3.65; 95% CI, 1.48-9.00), and each 10-point increase in preoperative pain was associated with a 17% increase in the odds of experiencing chronic pain (OR, 1.17; 95% CI, 1.02-1.34). Internal fixation was associated with decreased odds of developing chronic pain compared with the other 2 procedures (OR, 0.29; 95% CI, 0.10-0.90).

**Conclusions and Relevance:**

In this study, preoperative pain, time to surgery, and procedure type were modifiable factors associated with chronic pain 1 year after DRF treated with surgery. Adequate pain control in patients with acute DRFs even before definitive surgical management and earlier fixation for patients requiring surgery may decrease the risk of developing chronic pain. Internal fixation may decrease the risk of chronic pain after DRF surgery, compared with percutaneous pinning or external fixation.

**Trial Registration:**

ClinicalTrials.gov Identifier: NCT01589692

## Key Points

**Question:**

What are modifiable preoperative factors associated with developing chronic pain after distal radius fracture surgery?

**Findings:**

In this secondary analysis of a randomized clinical trial with 146 participants, each 10-point increase in preoperative pain score was associated with 17% increased odds of chronic pain, and a 1-week delay in surgical intervention was associated with more than triple the odds of experiencing chronic pain. Internal fixation was associated with decreased risk of chronic pain compared with external fixation or pinning.

**Meaning:**

In this study, earlier time to surgery, adequate preoperative pain control, and internal fixation were associated with lower risk of chronic pain development among patients with distal radius fracture who were treated surgically.

## Teaser

This secondary analysis identifies modifiable preoperative factors that are significantly associated with developing chronic pain among patients with distal radius fractures who are treated surgically.

## Introduction

Distal radius fracture (DRF) is a common injury, accounting for one-sixth of all fractures managed in the emergency department,^1,2,3,4^ with a lifetime risk of 33% in elderly women.^5^ As many as 63% of patients with DRF who receive appropriate treatment report some degree of persistent wrist pain 1 year after injury.^6^ Chronic musculoskeletal pain is among the leading health problems in older adults.^7,8^ It is associated with significant personal and societal burden because of psychosocial suffering, long-term analgesic use, loss of independence, and loss of productivity.^9^ Because of the high incidence of DRFs and the relatively high prevalence of patients with chronic pain after this injury, identifying modifiable preoperative factors that are associated with chronic pain could have considerable impact on patient care. In addition, given the ongoing deleterious effects of widespread opioid misuse despite efforts to optimize postoperative pain control, chronic pain reduction can decrease long-term narcotic use and addiction potential.

Pain is closely associated with patient-reported outcome scores after DRF management.^10^ Prior studies have shown medical comorbidities,^7^ injury compensation,^7,11^ and education level^7,11^ as contributing determinants of chronic pain after DRF. One study suggested education level and prereduction radial shortening to be associated with pain 6 months after DRF management.^11^ Pain catastrophizing has also been associated with finger stiffness after DRF.^12^ Conversely, characteristics of the injury, such as mechanism of injury, fall severity, or prereduction dorsal angulation, are not suggested to be associated with chronic pain.^7^ Radiological measurements also do not seem to be associated with pain because some patients with severe malunion have no chronic pain.^7^ However, the factors identified by previous studies are fixed patient factors that cannot be modified to alter outcomes. In addition, most studies have been performed at a single center and included limited follow-up data on patients. It is unknown whether modifiable preoperative factors in patients with DRF can help to prevent chronic pain following DRF surgery.

The aim of this study was to identify modifiable preoperative factors in patients with DRF associated with the likelihood of developing chronic pain after surgical management. To accomplish this, we performed a secondary analysis on data from the multicenter randomized Wrist and Radius Injury Surgical Trial (WRIST), with pain at 12 months after intervention as the primary outcome.

## Methods

### Study Design

**Figure 1.**
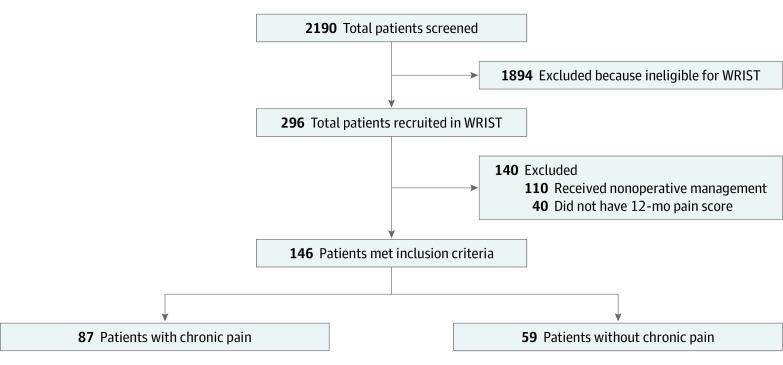
Flow Diagram of the Current Secondary Analysis Based on the Wrist and Radius Injury Surgical Trial (WRIST)

The Wrist and Radius Injury Surgical Trial (WRIST) is a multicenter randomized clinical trial of treatment for displaced, extra-articular DRFs in patients aged 60 years or older who were recommended for surgical fixation by participating surgeons based on clinical examination and radiographs. Participants in WRIST were enrolled from 24 sites in the United States, Canada, and Singapore from April 10, 2012, to December 31, 2016. Patients with open fractures, bilateral fractures, prior DRF on the same wrist, and concurrent severe trauma were excluded from the trial. For the current secondary analysis, all patients who received surgery and had 12-month pain assessments were included. Patients who did not undergo surgery were excluded to focus on postoperative chronic pain in DRFs (Figure 1). Detailed protocols of the study design, enrollment, study flow diagram, and results of WRIST have been illustrated previously (Supplement 1).^13^ Written consent was obtained from all participants, and the WRIST protocol was approved by each center. This study adhered to the Strengthening the Reporting of Observational Studies in Epidemiology (STROBE) reporting guideline. The current study was approved by the University of Michigan institutional review board.

### Study Variables

During the enrollment visit, within 1 week of injury, preoperative patient characteristics were collected. Potential patient factors associated with postoperative chronic pain were first identified using clinical judgment, as follows: age, number of comorbidities (ie, diabetes, hypertension, congestive heart failure, chronic obstructive pulmonary disease, and osteoarthritis), Rapid Assessment of Physical Activity score, presence of osteoarthritis, smoking history, education level, income, employment, 36-item Short Form (SF-36) physical component summary score, SF-36 mental component summary (MCS) score, and preoperative pain level. Perioperative variables including treatment type (volar locking plate internal fixation, external fixation, and percutaneous pinning), time to surgery from fracture, preoperative radial height, preoperative radial inclination, volar tilt, ulnar variance, presence of ulnar styloid fracture, AO classification, and reduction quality were assessed.

The Michigan Hand Outcomes Questionnaire (MHQ) pain domain was administered at baseline before surgery and 12 months after surgery as part of WRIST. The MHQ pain domain is scored from 0 to 100, with higher scores indicating more severe pain. It was previously validated for use among patients with DRF.^14^ The primary outcome was the difference in pain scores obtained by subtracting the pain score reported in the noninjured hand from the pain score reported in the injured hand. In accordance with the US Centers for Disease Control and Prevention (CDC) definition of chronic pain, which is pain lasting longer than 3 months or past the time of normal tissue healing,^15^ the primary outcome was categorized into a binary variable of chronic pain broadly as the presence of more severe pain in the injured hand compared with the uninjured hand 12 months after injury. As such, the presence of chronic pain was defined as an MHQ pain score difference greater than 0 between the injured and uninjured hand 1 year after surgery.

### Statistical Analysis

Because of concerns regarding potential selection bias from missingness in 12-month pain scores, we first divided the patients who met inclusion criteria into 2 groups (ie, those with and without 12-month pain scores) to assess the missing pattern and mechanism. We used *t* tests for continuous variables and χ^2^ tests for categorical variables to evaluate differences in each variable, comparing those patients with missing data to their counterparts. We fit a logistic regression model to estimate the probability of data completion for both groups and confirmed overlapping sampling probabilities between the 2 groups, indicating the potential for valid statistical analysis after adjustment. We used an inverse probability weighted (IPW) logistic regression for a binary outcome of presence or absence of chronic pain using the data with complete observations. Five covariates (ie, treatment type, sex, age, smoking status, and number of comorbidities) were forced to be included in the final model because of their known clinical implications. Subsequently, stepwise model selection was used to derive our final parsimonious multivariable regression model. We also used 5000 replications of bootstrapping with reestimated weights in each replicate to validate the inference using sandwich estimators in the IPW logistic regression. An a priori significance level was set at a 2-tailed *P* < .05. All statistical analyses were performed using RStudio version 1.2.5033 and R version 3.6.2 (R Project for Statistical Computing).

## Results

From a total of 296 patients in the WRIST database, 146 of the 186 patients who underwent surgery (78.5%) completed 12-month MHQ assessments. In the complete sample of 186 patients, the mean (SD) age was 68.4 (7.2) years, 110 (59.1%) were retired, 164 (88.2%) were women, and patients were relatively healthy, with a mean (SD) number comorbidities of 3.7 (2.7) (Table 1). There were slightly more individuals who did not smoke than those who did (98 [52.7%] vs 87 [46.8%]). In the subset of 146 patients with 12-month MHQ scores, the mean (SD) patient age was 68.9 (7.2) years, 128 (87.6%) were women, and 93 (63.7%) were retired. Overall, 87 of these patients (59.6%) reported chronic pain and 59 (40.4%) did not (Figure 1).

**Table 1.** Descriptive Statistics of Full Patient Cohort and Missing Data

Characteristic	Patients, No. (%)			*P* value^a^
	All (N = 186)	No 12-mo pain outcome (n = 40)	With 12-mo pain outcome (n = 146)	
Treatment type				
Volar locking plate	75 (40.3)	14 (35.0)	61 (41.8)	.79
External fixation	60 (32.3)	13 (32.5)	47 (32.2)	
Pinning	51 (27.4)	13 (32.5)	38 (26.0)	
Time to surgery from fracture, mean (SD), wk	0.5 (0.5)	0.6 (0.5)	0.5 (0.5)	NA
Age, mean (SD), y	68.4 (7.2)	66.7 (6.9)	68.9 (7.2)	.08
Sex				
Men	22 (11.8)	4 (10.0)	18 (12.4)	.93
Women	164 (88.2)	36 (90.0)	128 (87.6)	
RAPA score				
Active	82 (44.1)	13 (32.5)	69 (47.2)	.26
Underactive	85 (45.7)	22 (55.0)	63 (43.1)	
Sedentary	18 (9.7)	5 (12.5)	13 (8.9)	
Unknown	1 (0.5)	0 (0.0)	1 (0.7)	
Comorbidities, mean (SD), No.	3.4 (2.3)	3.7 (2.7)	3.3 (2.2)	.39
Osteoarthritis				
Present	66 (35.5)	12 (30.0)	54 (37.0)	.70
Absent	119 (64.0)	27 (67.5)	92 (63.0)	
Unknown	1 (0.5)	1 (2.5)	0 (0.0)	
Smoking				
Yes	87 (46.8)	23 (57.5)	64 (44.1)	.11
No	98 (52.7)	16 (40.0)	82 (55.9)	
Unknown	1 (0.5)	1 (2.5)	0 (0.0)	
Education				
<High school	19 (10.2)	6 (15.0)	13 (8.9)	.35
≥High school	161 (86.6)	32 (80.0)	129 (88.4)	
Unknown	6 (3.2)	2 (5.0)	4 (2.7)	
Income				
<$20 000	30 (16.1)	6 (15.0)	24 (16.4)	.91
$20 000-$60 000	79 (42.4)	18 (45.0)	61 (41.8)	
>$60 000	58 (31.2)	11 (27.5)	47 (32.2)	
Unknown	19 (10.2)	5 (12.5)	14 (9.6)	
Employment				
Retired	110 (59.1)	17 (42.5)	93 (63.7)	.10
Full time	58 (31.2)	15 (37.5)	43 (29.5)	
Other	18 (9.7)	8 (20.0)	10 (6.8)	
Dominant hand injury	76 (42.7)	10 (29.4)	66 (45.8)	.12
Preoperative radial height, mean (SD), mm	8.3 (3.6)	7.9 (4.3)	8.4 (3.4)	.53
Radial inclination, mean (SD), °	16.1 (5.3)	15.8 (7.6)	16.1 (5.3)	.81
Volar tilt, mean (SD), °	14.2 (13.9)	13.1 (15.1)	14.5 (13.6)	.61
Ulnar variance, mean (SD), mm	2.4 (2.9)	1.5 (2.6)	2.6 (3.0)	.04
Ulnar styloid fracture	75 (42.9)	10 (27.8)	65 (46.8)	.06
AO classification, No, (%)				
A	109 (58.6)	21 (52.5)	88 (60.3)	.53
C	67 (36.0)	16 (40.0)	51 (34.9)	
Unknown	10 (5.4)	3 (7.5)	7 (4.8)	
SF-36, mean (SD)				
PCS	34.1 (10.2)	33.2 (9.8)	34.3 (10.4)	.54
MCS	49.7 (13.8)	47.6 (13.4)	50.2 (13.9)	.30
Preoperative pain score, mean (SD)^b^	61.0 (27.6)	63.0 (27.8)	61.0 (27.7)	.63
Procedure difficulty^c^	4.3 (2.2)	4.1 (2.2)	4.3 (2.1)	.74
Reduction quality, mean (SD)^d^	7.7 (1.6)	7.6 (1.7)	7.8 (1.5)	.67

Abbreviations: NA, not applicable; MCS, mental component summary score; PCS, physical component summary score; RAPA, Rapid Assessment of Physical Activity; SF-36, 36-item Short Form.

^a^ Differences in means for continuous variables were assessed using *t* tests; association between baseline chronic pain and categorical variables was assessed using χ^2^ tests or Fisher exact test.

^b^ Determined by subtracting the uninjured hand Michigan Hand Outcomes Questionnaire pain domain score from the Michigan Hand Outcomes Questionnaire pain domain score of the injured hand within 1 week of injury.

^c^ Surgeons were asked to rate the difficulty of the procedure subjectively, with 1 being the easiest and 10 being the hardest.

^d^ Surgeons were asked to rate the reduction quality, with 0 being the worst quality reduction and 10 being the best quality reduction.

**Figure 2.**
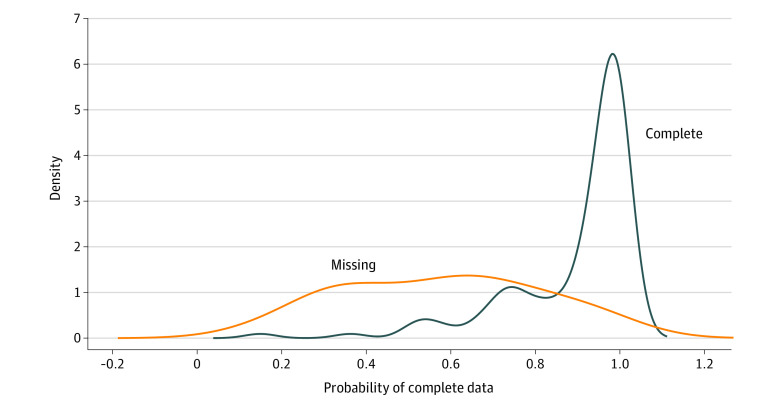
Probability of Complete Data in Patients With and Without 12-month MHQ Pain Scores The distributions have substantial difference, indicating possible selection bias. However, the degree of overlap suggests potential for valid statistical analysis after adjustment. MHQ indicates Michigan Hand Outcomes Questionnaire.

When comparing the patients with 12-month pain scores with those without, patients with missing pain scores had significantly lower ulnar positive variance (mean [SD], 1.5 [2.6] mm vs 2.6 [3.0] mm; *P* = .04) (Table 1). Despite not reaching statistical significance, some variables had contrasting missing patterns in the groups with and without 12-month pain scores. When compared with the cohorts with complete data, patients with missing pain scores were more likely younger (mean [SD] age, 66.7 [6.9] years vs 68.9 [7.2] years; *P* = .08), working full-time (15 [37.5%] vs 43 [29.5%]; *P* = .10), and without ulnar styloid fractures (26 [72.0%] vs 74 [53.0%]; *P* = .06), but these differences were not statistically significant. These trends demonstrated that the observed data may lead to selection bias. A multivariable logistic regression of all covariates showed that lower education level, working full-time, and less ulnar positive variance were statistically significantly associated with missingness in 12-month pain scores (Table 2). The distribution of sampling probabilities between the groups with and without 12-month pain scores varied substantially but overlapped well (Figure 2). To account for such differences in patients with and without 12-month pain outcomes, an IPW logistic regression was fitted for analysis.

**Table 2.** Logistic Regression of Covariates and Missingness in Data

Factor	Estimate (SE)^a^	Odds ratio (95% CI)	*P* value
Treatment type			
Pinning	−0.57 (0.75)	0.57 (0.13 to 2.46)	.45
Volar locking plate	−1.23 (0.83)	0.29 (0.06 to 1.49)	.14
Male sex	−0.06 (0.88)	0.94 (0.17 to 5.30)	.95
RAPA score			
Sedentary	−0.02 (1.85)	0.98 (0.03 to 36.93)	.99
Underactive	0.65 (0.85)	1.92 (0.36 to 10.16)	.44
Osteoarthritis present	−1.33 (1.09)	0.27 (0.03 to 2.24)	.22
Smoking status present	0.67 (0.51)	1.95 (0.72 to 5.31)	.19
≥High school education	−3.32 (1.36)	0.04 (0 to 0.52)	.02
Income			
>$60 000	0.73 (1.29)	2.07 (0.17 to 25.96)	.57
$20 000-$60 000	0.67 (1.09)	1.95 (0.23 to 16.52)	.54
Retired	−1.91 (0.70)	0.15 (0.04 to 0.59)	.01
Dominant hand injury	−0.62 (0.70)	0.54 (0.14 to 2.13)	.38
Ulnar styloid fracture	−1.45 (0.67)	0.23 (0.06 to 0.87)	.03
AO classification, type C	1.23 (0.79)	3.42 (0.73 to 16.09)	.12
Time to surgery from fracture, wk	0.74 (0.65)	2.10 (0.59 to 7.51)	.25
Age	−0.06 (0.06)	0.94 (0.84 to 1.06)	.32
Comorbidities, No.	0.07 (0.14)	1.07 (0.81 to 1.40)	.65
Preoperative radial height, mm	0.01 (0.14)	1.01 (0.77 to 1.33)	.95
Radial inclination, °	−0.06 (0.10)	0.94 (0.77 to 1.14)	.51
Volar tilt, °	−0.03 (0.02)	0.97 (0.94 to 1.01)	.24
Ulnar variance	−0.54 (0.20)	0.59 (0.40 to 0.87)	.01
SF-36			
PCS	0.01 (0.03)	1.01 (0.95 to 1.07)	.86
MCS	0.03 (0.03)	1.03 (0.97 to 1.09)	.30
Baseline pain score^b^	0.23 (0.16)	1.25 (0.92 to 1.72)	.17
Procedure difficulty^c^	−0.07 (0.17)	0.93 (0.67 to 1.30)	.66
Reduction quality^d^	−0.07 (0.22)	0.94 (0.61 to 1.44)	.76

Abbreviations: MCS, mental component summary score; PCS, physical component summary score; RAPA, Rapid Assessment of Physical Activity; SF-36, 36-item Short Form.

^a^ Sandwich estimate.

^b^ Determined by subtracting the uninjured hand Michigan Hand Outcomes Questionnaire pain domain score from the Michigan Hand Outcomes Questionnaire pain domain score of the injured hand.

^c^ Surgeons were asked to rate the difficulty of the procedure subjectively, with 1 being the easiest and 10 being the hardest.

^d^ Surgeons were asked to rate the reduction quality, with 0 being the worst quality reduction and 10 being the best quality reduction.

The following covariates were selected for inclusion in the final IPW logistic model: treatment type, age, sex, smoking status, number of comorbidities, time to surgery, preoperative volar tilt, preoperative SF-36 MCS score, and preoperative pain. Variables found to have a statistically significant association with chronic pain in the multivariable model were time to surgery, preoperative pain, and treatment with volar locking plate (Table 3). For each day delayed from fracture until surgery, there was a 17% (odds ratio [OR], 1.17; 95% CI, 1.00-1.37; *P* = .002) increased odds of developing chronic pain postoperatively. Delaying surgery by 1 week was associated with approximately 3.7-fold higher odds of developing chronic pain compared with performing surgery on the day of injury (OR, 3.65; 95% CI, 1.48-9.00; *P* = .004). Preoperative pain was also found to be significantly associated with chronic postoperative pain. An increase of 10 points in the MHQ pain domain was associated with a 17% (OR, 1.17; 95% CI, 1.02-1.34; *P* = .04) increased odds of developing chronic pain. Treatment with volar locking plate was associated with 71% decreased odds (OR, 0.29; 95% CI, 0.10-0.90; *P* = .03) of developing chronic pain compared with external fixation and 77% lower odds (OR, 0.23; 95% CI, 0.08-0.72; *P* = .006) of developing chronic pain compared with percutaneous pinning. The full model including all covariates had a residual deviance of 141.90, while the reduced model after variable selection had a residual deviance of 153.15. The validation results from bootstrapping were nearly identical to that of the main model (eTable in Supplement 2). Hypothesis testing with a likelihood ratio test revealed no significant difference between the full model and the reduced model; thus, the reduced model was selected. The final model was checked for collinearity to ensure model stability and outliers.

**Table 3.** Inverse Probability Weighted Logistic Regression

Factor	Estimate (SE)^a^	Odds ratio (95% CI)	*P* value
Treatment type			
Pinning	0.35 (0.57)	1.42 (0.46 to 4.34)	.54
Volar locking plate	−1.22 (0.57)	0.29 (0.10 to 0.90)	.03
Male sex	1.30 (0.67)	3.67 (0.99 to 13.66)	.051
Smoking status present	0.85 (0.46)	2.33 (0.95 to 5.74)	.06
≥High school education	−1.16 (0.82)	0.31 (0.06 to 1.56)	.16
Time to surgery from fracture, wk	1.30 (0.46)	3.65 (1.48 to 9.00)	.004
Age	0.008 (0.03)	1.01 (0.95 to 1.07)	.81
Comorbidities, No.	−0.017 (0.10)	0.98 (0.81 to 1.20)	.87
Volar tilt, °	−0.024 (0.02)	0.98 (0.94 to 1.02)	.12
SF-36 MCS	−0.028 (0.02)	0.97 (0.94 to 1.01)	.09
Preoperative pain score^b^	0.158 (0.07)	1.17 (1.02 to 1.34)	.03

Abbreviations: MCS, mental component summary score; SF-36, 36-item Short Form.

^a^ Sandwich estimate.

^b^ Determined by subtracting the uninjured hand Michigan Hand Outcomes Questionnaire pain domain score from the Michigan Hand Outcomes Questionnaire pain domain score of the injured hand (parameter for every 10-point increase in Michigan Hand Outcomes Questionnaire pain score).

## Discussion

In this secondary analysis of prospectively collected data from a randomized clinical trial, we studied preoperative modifiable factors associated with chronic hand and wrist pain among patients with DRF 1 year after surgery. Approximately 60% of our cohort reported some degree of persistent pain 1 year after DRF surgery, which is consistent with rates reported in the literature.^6^ We found that delayed time to surgery from fracture, higher preoperative pain levels, and treatment with external fixation or percutaneous pinning were significantly associated with chronic postoperative pain after DRF.

The results of our analysis differ from previous studies that used regression modeling to estimate rates of chronic pain. Grewal et al^7^ identified third-party compensation claim, education level, and comorbidities as associated with chronic pain 1 year after extra-articular DRF. Although not reaching statistical significance, the only patient factors that may have been associated with chronic pain in our study were positive smoking status and male sex. This supports prior findings that individuals who smoke are more likely to be men and require higher postoperative narcotic use after undergoing general anesthesia.^16,17^ Another study with a smaller cohort found that prereduction radial shortening and education level were significantly associated with 6-month pain after DRF.^11^ Based on our analysis, these were not associated with chronic pain and were not selected for inclusion in the multivariable model. In addition, these covariates are not modifiable baseline factors that could be optimized preoperatively. This contrasting finding may be because prior studies were single-center studies, whereas WRIST is an international multicenter study enrolling patients from various geographical and practice settings. Also, the current analysis exclusively studied patients who underwent surgery after DRF, whereas past studies included patients who were treated nonoperatively.

Approximately 22% (40 of 186 patients) of the operative cohort was missing 12-month pain scores, and after adjusting for covariates using a multivariable logistic regression, lower education level, patients who were working full time, and less ulnar positive variance were associated with missing data. It is conceivable that patients working full time were more likely to be lost to follow-up because of their work schedule compared with retirees. Similarly, patients with lower education levels may have had occupations that would not allow abundant time off to attend follow-up appointments. On the other hand, greater ulnar variance, possibly indicating more severely impacted DRFs, may have resulted in more diligent follow-up visits. To account for this potential nonrandom missingness, an IPW model was used for the final regression.

Our analysis identified preoperative pain as a variable associated with chronic pain 1 year after DRF. This finding lends support to a previous analysis that described baseline pain as associated with chronic pain after nonoperative DRF management.^18^ The mechanism of baseline pain translating to persistent chronic pain remains unclear but, based on our results, appears to be independent of age, medical comorbidities, and treatment type. One potential explanation pertains to psychosocial aspects, including pain catastrophizing, which is shown to be associated with pain and disability after musculoskeletal trauma.^20^ Another possible explanation is pain centralization. Centralized pain syndromes, such as fibromyalgia, are thought to occur from a combination of pathways, including increased peripheral input via nociceptor activation leading to long-term potentiation in the central nervous system.^21^ Conceivably, poorly controlled pain after DRF may hypersensitize the peripheral and central nervous systems to become more susceptible to developing chronic pain. Furthermore, a prediction model based on a prospective study of DRFs indicated that patients with baseline pain scores greater than 5 of 10 were at significantly higher risk of developing complex regional pain syndrome (CRPS) 4 months after fracture.^22^ This implies that adequate management of pain and/or psychosocial factors during and after the initial reduction of DRF in the emergency department with nonnarcotic oral pain medications may decrease the incidence of DRF-related chronic pain and possibly CRPS.

Another modifiable factor associated with chronic pain was time to surgery. If the patient requires surgery, most surgeons prefer surgical fixation within 2 weeks of injury, before substantial callus formation.^23^ However, our findings suggest that earlier time to surgery may be associated with decreased odds of developing chronic pain. The explanation behind this finding is likely multifactorial. It may be that earlier anatomic reduction of the radius may mitigate pain by decreasing pain centralization, or it might be related to earlier hand and occupational therapy interventions that may have a protective effect against chronic pain.^24^ The wide variation of time to surgery was a surprising finding and may imply access to care barriers for some patients with DRF. Future studies are warranted to elucidate the obstacles to timely DRF care.

The association between volar locking plate internal fixation and decreased chronic pain development compared with percutaneous pinning or external fixation was an unexpected finding. Prior meta-analyses comparing internal fixation to external fixation and percutaneous pinning concluded that there were no differences in pain outcomes among the treatment types.^25,26^ But 1 study^26^ suggested that there was an association with higher incidence of CRPS after percutaneous pinning than internal fixation. It is unclear why internal fixation is associated with decreased incidence of chronic pain compared with other surgical treatments. One potential explanation may be that visible hardware outside of the skin in percutaneous pinning and external fixation, unlike internal fixation, may increase pain catastrophizing. Pain catastrophizing has been identified as a significant factor associated with chronic pain in musculoskeletal surgery.^27,28^ We are not suggesting that all patients who had chronic pain in our study eventually progressed to CRPS given that it is reported that the incidence of CRPS is between 3.8% to 7.0% after fracture.^22,29^ However, among the most common triggers of CRPS is DRF,^30^ with a reported annual lost income of $1 billion dollars per year^31^; therefore, prevention of such a devastating complication is an important topic in fracture management that requires attention.

This study benefits from a multicenter international randomized clinical trial database that included patients in private and academic centers, increasing its generalizability. Also, unlike in some previous studies, this study only included patients with DRF who received operative care. Furthermore, to our knowledge, this is among the few studies to investigate modifiable preoperative and perioperative factors in patients with DRF designed to help prevent the onset of chronic pain.

### Limitations

Several limitations must be considered while interpreting these results. Although this study is based on a prospective multicenter randomized clinical trial, there may be other modifiable preoperative factors that were not collected. One such parameter may be more specific psychosocial data and risk factors for psychological distress, such as prior opioid use, which were not primary endpoints of WRIST. In addition, slightly less than one-quarter of our initial cohort did not have 12-month follow-up pain assessments and were excluded, leading to possible selection bias. To mitigate the effects of missing data, we used an IPW model for analysis. Because patients were recruited from 24 study sites, there is potential for heterogeneity; however, we attempted to minimize heterogeneity with strict inclusion and exclusion criteria during recruitment. Lastly, because we defined chronic pain as pain lasting longer than 3 months or past the time of normal tissue healing in accordance with the CDC, we could not differentiate patients with functionally debilitating chronic pain from those with mild chronic pain.

## Conclusions

In this study, 3 modifiable factors—lower preoperative pain, decreased time to surgery, and internal fixation—were associated with reduced incidence of chronic pain 1 year after DRF surgery. Health care professionals should be mindful of acute pain even before definitive surgical intervention to ensure optimal long-term outcomes after DRF management. Patients with acute DRFs should have sufficient pain control with nonnarcotic analgesics and appropriately molded splint immobilization before undergoing definitive surgery. Unstable DRFs requiring surgery may benefit from undergoing fixation as early as possible to decrease the probability of chronic pain development. Internal fixation may confer some protective effects against chronic pain compared with external fixation or percutaneous pinning, but further validation studies are warranted before any specific practice recommendations are made. Given the prevalence of DRFs in the elderly and the ongoing opioid crisis, chronic pain prevention should be a focus in all musculoskeletal surgery for improved patient-reported outcomes and function.

## Notes

Supplement 1. **Trial Protocol**

Supplement 2. **eTable.** Inverse Probability Weighted Logistic Regression from Bootstrapping

Supplement 3. **Data Sharing Statement**
