# Supplementary material for: Modifiable Factors Associated With Chronic Pain 1 Year After Operative Management of Distal Radius Fractures: A Secondary Analysis of a Randomized Clinical Trial
Source: JAMA Netw Open. 2020 Dec 18;3(12):e2028929. doi: 10.1001/jamanetworkopen.2020.28929 (PMC7749439; doi:10.1001/jamanetworkopen.2020.28929)
Supplement: Supplement 2. — eTable. Inverse Probability Weighted Logistic Regression from Bootstrapping [file jamanetwopen-e2028929-s002.pdf]

Supplemental Online Content

Yoon AP, Wang C, Speth KA, Wang L, Chung KC; The WRIST Group. Modifiable factors associated with chronic pain 1 year after operative management of distal radius fractures: a secondary analysis of a randomized clinical trial. *JAMA Netw Open*. 2020;3(12):e2028929. doi:10.1001/jamanetworkopen.2020.28929

eTable. Inverse Probability Weighted Logistic Regression from Bootstrapping

This supplemental material has been provided by the authors to give readers additional information about their work.

© 2020 Yoon AP et al. *JAMA Network Open.*

eTable. Inverse Probability Weighted Logistic Regression from Bootstrapping			
Predictor	Estimate (Standard Error)	Odds Ratio (95% CI)	Empirical P-value
Treatment Type			
Pinning	0.35 (0.56)	1.42 (0.48,4.17)	0.56
Volar Locking Plate	-1.22 (0.54)	0.29 (0.11,0.81)	0.03*
Gender (male)	1.30 (0.65)	3.67 (0.99,13.66)	0.05
Smoking status (yes)	0.85 (0.45)	2.33 (0.98,5.52)	0.06
Education (above high school)	-1.16 (0.95)	0.31 (0.06,1.59)	0.29
Time to Surgery from Fracture (weeks)	1.30 (0.45)	3.65 (1.60,8.32)	0.003*
Age	0.008 (0.03)	1.01 (0.95,1.07)	0.81
Number of Comorbidities	-0.017 (0.10)	0.98 (0.81,1.20)	0.91
Volar tilt (degrees)	-0.024 (0.02)	0.98 (0.94,1.02)	0.11
SF-36 MCS	-0.028 (0.02)	0.97 (0.94,1.01)	0.10
Baseline Pain Score[†]	0.158 (0.07)	1.17 (1.00,1.37)	0.03*
* Statistical Significance			
[†] Determined by subtracting the uninjured hand MHQ pain domain score from the MHQ pain domain score of the injured hand			

© 2020 Yoon AP et al. *JAMA Network Open.*
