# Supplementary material for: Modifiable Factors Associated With Chronic Pain 1 Year After Operative Management of Distal Radius Fractures: A Secondary Analysis of a Randomized Clinical Trial
Source: JAMA Netw Open. 2020 Dec 18;3(12):e2028929. doi: 10.1001/jamanetworkopen.2020.28929 (PMC7749439; doi:10.1001/jamanetworkopen.2020.28929)
Supplement: Supplement 3. — Data Sharing Statement [file jamanetwopen-e2028929-s003.pdf]

Data Sharing Statement

Yoon. Modifiable Factors Associated With Chronic Pain 1 Year After Operative Management of Distal Radius Fractures. *JAMA Netw Open*. Published December 18, 2020.
doi:10.1001/jamanetworkopen.2020.28929

Data
Data available: No

Additional Information
Explanation for why data not available: The data can be available upon request should the manuscript be accepted
